# Clustering of lifestyle factors and the relationship with mental health among college students: a latent profile analysis

**DOI:** 10.1186/s12889-025-24501-6

**Published:** 2025-09-30

**Authors:** Gusonghan Maitiniyazi, Maierhaba Dilimulati, Remila Abulaiti, Zulihuma Nijiati, Nazhakaiti Tuergong, Ting Ma, Chunpeng Li, Shufang Xia

**Affiliations:** 1 School of Nursing, Xinjiang Hetian College, Hetian, Xinjiang China; 2 Xinjiang Key Laboratory of Hetian Characteristic Chinese Traditional Medicine Research, Hetian, Xinjiang China; 3 Wuxi School of Medicine, Jiangnan University, Wuxi, Jiangsu China

**Keywords:** Lifestyle, Mental health, College students, Depression, Anxiety, Latent profile analysis

## Abstract

**Background:**

While substantial evidence links single lifestyle factors to mental health, research on how multiple co-occurring healthy lifestyle behaviors relate to mental health, particularly among college students, remains limited. This study aimed to identify distinct profiles of healthy lifestyle behaviors and examine their associations with mental health among college students.

**Method:**

This cross-sectional study included 1340 college students (mean age = 19.4 years; *SD* = 1.2). Self-reported data were collected on diet, sleep, physical activity (PA), screen time, and sedentary behavior. Mental health was assessed using the Depression, Anxiety and Stress Scale-21 (DASS-21). Latent profile analysis was used to identify groups with similar lifestyle characteristics. Multiple linear regression was applied to examine associations between lifestyle profiles and mental health.

**Result:**

Three lifestyle profiles were identified: “Active Engagement” (*n* = 520, 38.8%), “Moderate Engagement” (*n* = 478, 35.7%), and “Negative Engagement” (*n* = 342, 25.5%). These groups showed significant differences in anxiety, depression and stress levels (*p* < 0.001). Monthly family income and sex predicted profile membership. Compared with the “Active Engagement” group, the “Moderate Engagement” and “Negative Engagement” groups showed a higher risk of mental health problems.

**Conclusions:**

Our findings suggest that interventions specifically targeting the active lifestyle pattern (characterized by the higher PA, low sedentary behavior and screen time) may be particularly effective for improving mental health in college students, moving beyond simply promoting single health behaviors.

## Introduction

Mental health problems, such as anxiety, depression, and stress, are major public health concerns and are highly prevalent among the college-aged population [1, 2]. A study of students from 15 universities in China reported that 32.0%, 43.0%, and 26.0% of freshmen exhibited symptoms of depression, anxiety, and stress, respectively [3]. Poor mental health during this developmental stage can cause a decline in academic performance, sleep deprivation [4], and even suicidal behavior [5]. These adverse emotional states may have persistent effects and significantly influence individuals throughout their lives. Current mental health education initiatives for college students face multiple challenges, including insufficient attention, a shortage of qualified educators, superficial implementation, limited channels, incomplete systems, and low relevance to students’ actual needs [6]. Therefore, college students encounter substantial mental health challenges, highlighting the necessity for further research into symptoms such as depression, anxiety, and stress.

The determinants of college students’ mental health are lied in the dynamic interaction of biological, psychological, and social factors, as posited by the Biopsychosocial Model [7, 8]. Within this framework, as core components of daily routines, lifestyle behaviors emerge as pivotal psychosocial determinants of psychological well-being. To date, research on the association between college students’ lifestyle and mental health has largely focused on single behaviors (e.g., diet or physical activity (PA)), despite the Biopsychosocial model emphasizing the impact of multi-dimensional lifestyles on mental health. Previous studies have identified widespread lifestyle issues among college students, such as unhealthy dietary habits [9], sedentary behavior [10], insufficient PA, and poor sleep quality [11]. High-quality sleep, regular PA, and a balanced diet have been shown to positively affect mental health in this population [5, 12, 13]. Despite an increasing awareness of the significance of fostering healthy lifestyle practices to support mental health, several critical inquiries remain unresolved. Foremost among these is the degree to which the aggregation of multiple lifestyle behaviors correlates with symptoms of depression, anxiety, and stress. While it is well-documented that each individual healthy lifestyle behavior is linked to mental health, the extent to which multiple such behaviors coexist and relate to psychological well-being outcomes remains less understood.

Latent profile analysis (LPA) is a statistical approach that can identify individuals with similar pattern of lifestyle health behaviors that are associated with optimal mental health maintenance [14]. This method can enhance precise selection of specific evidence-based interventions that will target only those lifestyle health behaviors that are associated with optimal mental health and are deficient for the individual participant. A systematic review and meta-analysis demonstrated that individuals engaging in the healthiest clusters of lifestyle behaviors reported significantly fewer symptoms of depression, anxiety and psychological distress compared with those engaging in less healthy combinations of lifestyle behaviors [15]. Considering that LPA facilitates the classification of lifestyle behavioral patterns in college student populations and reveals the interactions between behavioral variables in different subgroups, it can provide new insights into how multidimensional lifestyle factors work together to influence the mental health of college students. Therefore, the aims of this study are to identify sub-groups, or profiles, of individuals based on lifestyle-related behaviors (PA, diet, sleep, sedentary behaviors, and screen time) and to examine associations between these profiles and mental health in Chinese college students.

## Materials and methods

### Study design and participants

This observational cross-sectional study was conducted from September to December 2023 to investigate potential associations between lifestyle factors and mental health status among college students at a single college in China. The survey was administered anonymously via WeChat, a major social media platform in China. The final analytical sample comprised 1340 college students aged 17–23 years. Before completing the questionnaire, all participants provided electronic informed consent after receiving a comprehensive explanation of the study objectives and procedures. The study was performed in accordance with the ethical principles outlined in the Declaration of Helsinki.

### Measurement

#### Mental health assessment

Mental health was assessed using the 21-item Depression, Anxiety and Stress Scale (DASS-21) [16], a validated instrument comprising three subscales: depression (7 items), anxiety (7 items), and stress (7 items). Each item was scored on a 4-point Likert scale (0 = *did not apply to me*, 3 = *I have been doing this a lot*). The total score for each subscale ranged from 0 to 42. Subscale scores were summed and multiplied by 2 to yield indices for stress, anxiety, and depression. Higher scores on each subscale indicate greater symptom severity. The internal consistency indices (Cronbach’s alpha) for the Chinese version of the DASS-21 were excellent (overall = 0.95; depression = 0.87; anxiety = 0.84; and stress = 0.88) in this study.

#### Physical activity assessment

PA was quantified using metabolic equivalent (MET) values from the International Physical Activity Questionnaire Short Form (IPAQ-SF), a validated instrument with demonstrating established reliability across heterogeneous populations [17]. Participants retrospectively reported daily durations of vigorous- and moderate-intensity PA during the preceding seven days according to standardized IPAQ-SF scoring protocols.

#### Diet assessment

Diet was evaluated using the Dietary Quality Questionnaire (DQQ) [18], a validated instrument comprising 29 yes/no items assessing consumption of sentinel food groups during the previous 24 h. The DQQ has been adapted for represent foods in the Chinese context that could reliably capture the food group consumption for the Chinese population, and the identification of sentinel food items for China has been described elsewhere. The China DQQ framework and scoring protocols are publicly accessible through the Global Diet Quality Project platform. In this study, the Cronbach’s alpha was 0.73.

#### Sleep assessment

Sleep was assessed using the 7-item Insomnia Severity Index (ISI), a self-administered questionnaire utilizing a 5-point Likert scale ranging from 0 (indicating minimal or no insomnia) to 4 (indicating significant or severe problems with insomnia). Item scores were summed to generate a total score ranging from 0 to 28, with higher scores reflecting greater insomnia severity. The ISI has demonstrated good psychometric properties [19, 20]. In this study, the Cronbach’s alpha was 0.90.

#### Screen time assessment

Screen time was measured through purpose-designed questions assessing daily time spent on electronic devices, including television, video games, as well as computers, and mobile phones. Average screen time was calculated using the following formula: ([weekday screen time × 5] + [weekend screen time × 2]) ÷ 7 [21].

#### Sedentary behavior assessment

Sedentary behavior was assessed using a single self-report item from the IPAQ-SF [17], in which participants reported the time (hours and minutes) spent sitting during the previous seven days.

### Statistical analysis

LPA was conducted in MPLUS 8.3 to identify profiles among lifestyle behaviors, and a varying number of groups were explored (1–5 models). All five factors (screen time, sleep, sedentary behavior, PA and diet) were standardized using Z-scores prior to LPA. The optimal number profiles was determined by incrementally increasing the number of groups and evaluating information criteria, including the Akaike information criterion (AIC), Bayesian information criterion (BIC), adjusted BIC (a-BIC), classification quality (entropy), and model comparison verification (Lo–Mendell–Rubin likelihood ratio tests [LMR-LRT] and bootstrapped likelihood ratio test [BLRT]). Lower AIC, BiC, and a-BIC values indicate better model fit. Entropy ranges from 0 to 1, with higher values (> 0.8) indicating clearer group separation. When LMR-LRT and BLRT *p*-values were significant, the k-1 model was rejected and the k-profile model was selected.

SPSS 27.0 was used to analyze differences in sociodemographic characteristics and lifestyle behaviors across profiles determined by LPA. Shapiro–Wilk test, quantile–quantile (Q–Q) plots, and visual checking of histograms were used to confirm the normal distribution of variables. Chi-square (χ²) tests and one-way ANOVA were applied to compare sociodemographic and lifestyle variables across profiles. Logistic regression analysis was performed to assess the effects of various factors on latent profiles. Multiple linear regression analysis was subsequently used to examine associations between lifestyle behavior classification and DASS-21 scores, as well as scores for each subscale. Model assumptions were examined graphically and analytically and found to be adequately met. A *p* < 0.05 was considered statistically significant.

## Results

### Latent profile of lifestyles among participants

**Fig. 1:**
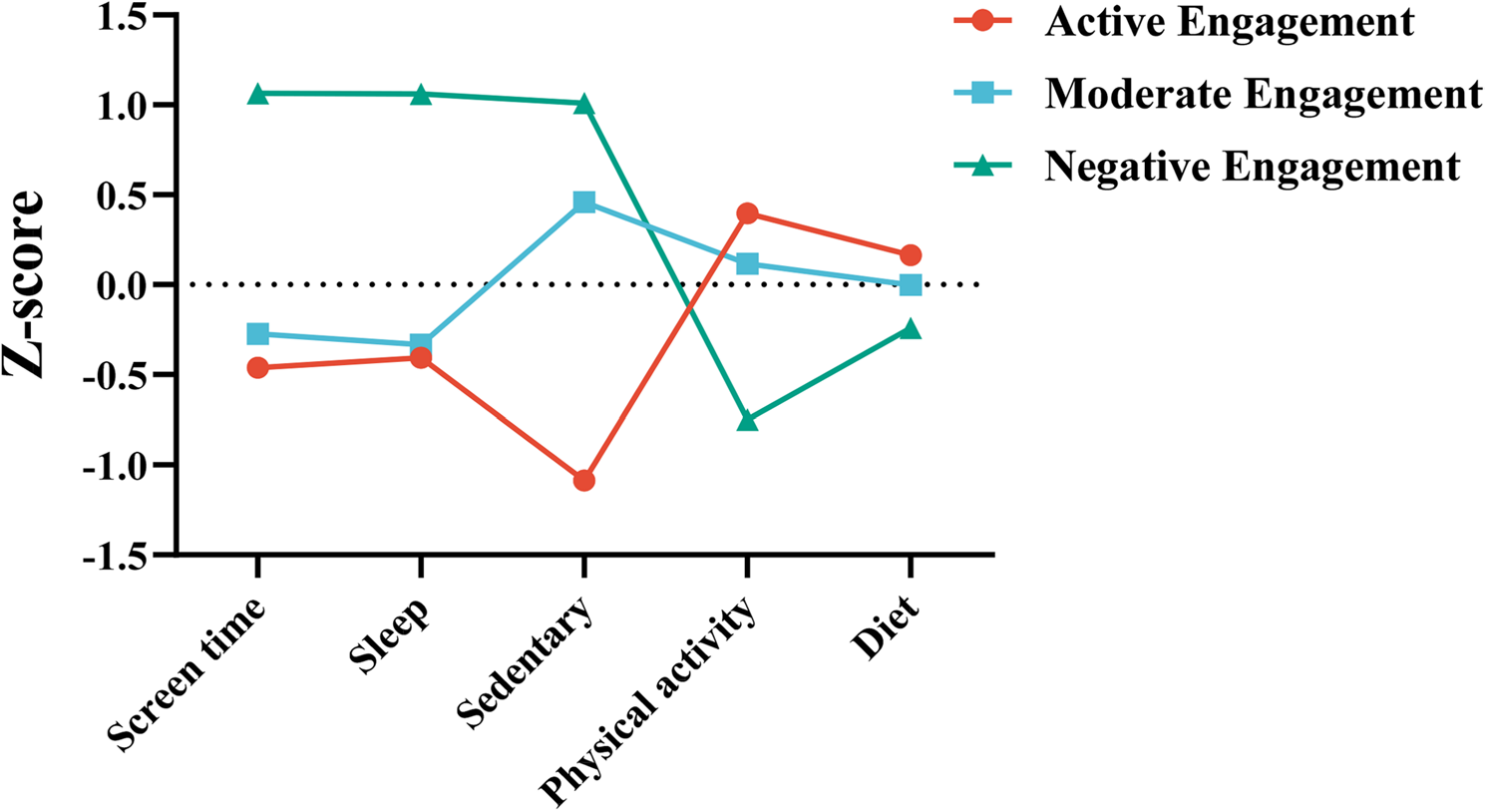
Latent profiles of college students’ lifestyles. Colored lines represent average z-scores for each lifestyle behaviors across each profile

Five LPA models were constructed to explore lifestyle profiles based on screen time, sleep, sedentary behavior, PA, and diet. The model fit statistics are summarized in Table 1. All models exhibited acceptable entropy values (> 0.80), indicating reliable profile separation. The 3-profile solution demonstrated the highest entropy, indicating the clearest group differentiation. While AIC, BiC, and a-BIC improved monotonically with more profiles, reflecting better statistical fit, we prioritized a holistic evaluation that integrated theoretical interpretability and parsimony. Critically, the 3-profile model struck the strongest balance: its entropy was the highest among all solutions, and additional profiles (4–5) introduced only marginal decreases in AIC/BIC/a-BIC while risking overfitting and diluting substantive meaning—subgroups in higher-profile models showed increasingly overlapping lifestyle patterns (as corroborated by profile mean comparisons, see Table 1). After assessing profile proportions (ranging from 25.5 to 38.8% for the 3-profile solution, avoiding overly small or unmeaningful subgroups) and the distinctness of their behavioral/psychological characteristics, we selected the 3-profile model for subsequent analyses. As shown in Fig. 1, the three identified profiles were: “Active Engagement” (Profile 1, 38.8%), “Moderate Engagement” (Profile 2, 35.7%), and “Negative Engagement” (Profile 3, 25.5%). Each profile reflected unique lifestyle and health associations aligned with the study objectives.

**Table 1:** Model fit statistics for each of the fitted latent profile analysis (LPA) models (*n* = 1340)

Number of profiles	AIC	BIC	a-BIC	Entropy	LMR-LRT (*p*)	BLRT (*p*)	Class proportion (%)
1	19028.774	19080.779	19049.013	-	-	-	1.000
2	17992.642	18075.849	18025.024	0.818	<0.001	<0.001	0.702/0.298
3	17578.410	17692.819	17622.935	0.825	<0.001	<0.001	0.389/0.352/0.259
4	17494.386	17639.997	17551.054	0.817	<0.001	<0.001	0.341/0.259/0.155/0.245
5	17318.420	17495.235	17387.232	0.817	<0.001	<0.001	0.239/0.259/0.100/0.250/0.152

*AIC* Akaike information criterion; *BIC* Bayesian information criterion; *a-BIC* adjusted BIC; *LMR-LRT* Lo-Mendell-Rubin-likelihood ratio test; *BLRT* Bootstrapped-likelihood ratio test

### Different characteristics between lifestyle profiles

The estimates for the basic characteristics and lifestyle behaviors (means for continuous variables and response probabilities for categorical variables) within each of the three profiles are summarized in Table 2. Statistically significant differences were observed among profiles for sex, family monthly income, smoking, alcohol use, screen time, diet, sleep, PA, sedentary behavior, depression, anxiety, and stress levels among the three profiles (all *p* < 0.05).

**Table 2:** Sample characteristics of each lifestyle profile (*n* = 1340)

Variable		Active Engagement (*n* = 520)	Moderate Engagement (*n* = 478)	Negative Engagement (*n* = 342)	F/χ^2^	*p*
Age (years)		19.5 ± 1.2	19.4 ± 1.1	19.4 ± 1.2	2.049	0.129
BMI (kg/m^2^)		20.9 ± 3.2	20.7 ± 2.8	21.1 ± 3.3	1.894	0.151
Sex						
Female		299 (57.5%)	320 (66.9%)	204 (59.6%)	9.984	0.007
Male		221 (42.5%)	158 (33.1%)	138 (40.4%)		
Family monthly income (RMB)						
< 2000		297 (57.1%)	293 (61.3%)	234 (68.4%)	14.236	0.007
2000~5000		189 (36.3%)	165 (34.5%)	98 (28.7%)		
>5000		34 (6.5%)	20 (4.2%)	10 (2.9%)		
Being an only child						
Yes		55 (10.6%)	52 (10.9%)	35 (10.2%)	0.088	0.957
No		465 (89.4%)	426 (89.1%)	307 (89.8%)		
Residence						
Urban		99 (19.0%)	82 (17.2%)	63 (18.4%)	0.607	0.738
Rural		421 (81.0%)	396 (82.8%)	279 (81.6%)		
Smoking						
Yes		10 (1.9%)	13 (2.7%)	17 (5.0%)	6.798	0.033
No		510 (98.1%)	478 (97.3%)	325 (95.0%)		
Alcohol use						
Yes		12 (2.3%)	13 (2.7%)	18 (5.3%)	6.375	0.041
No		508 (97.7%)	465 (97.3%)	324 (94.7%)		
Screen time (h/day)		4.7 ± 1.8	5.1 ± 1.6	8.0 ± 1.4	475.258	< 0.001
Diet		10.0 ± 4.7	9.3 ± 4.1	8.3 ± 3.7	18.025	< 0.001
Sleep		4.9 ± 4.3	5.1 ± 3.9	13.1 ± 4.2	492.210	< 0.001
PA (MET/min/week)		2259.2 ± 1002.0	2016.8 ± 921.8	1093.9 ± 554.8	191.234	< 0.001
Sedentary behavior (min/day)		128.9 ± 66.4	414.5 ± 86.8	516.9 ± 74.6	3124.133	< 0.001
Mental health						
Depression		1.8 ± 3.2	2.9 ± 4.0	12.0 ± 5.7	695.151	< 0.001
Anxiety		2.1 ± 3.3	3.7 ± 4.1	12.1 ± 5.3	642.060	< 0.001
Stress		2.1 ± 3.6	3.7 ± 4.4	13.0 ± 5.5	687.475	< 0.001

The result in the table is *n* (%) or mean ± standard deviation. *BMI* Body mass index; *PA* Physical activity

### Factors influencing latent lifestyle profiles in college students

To determine whether participant characteristics were associated with the lifestyle profiles, we put the three latent profiles were as the dependent variables. Variables showing significant (*p* < 0.05) group differences (sex, monthly family income, smoking, and alcohol use) in chi-square tests were entered as independent variables. Using “Active Engagement” as the reference, multiple logistic regression analysis was conducted with gender (males as reference), monthly family income (> 5000 as reference), smoking (“no” as reference), and alcohol use (“no” as reference) as independent variables (Table 3). Compared with male students, females were more likely to be in the “Moderate Engagement” group (odds ratio [OR] = 1.527; 95% confidence interval [CI]: 1.174–1.985; *p* = 0.002). Compared to students with a family monthly income > 5000, those with < 2000 was more likely to be in the “Negative Engagement” group (OR = 2.937; 95% CI: 1.404–6.144; *p* = 0.004).

**Table 3:** The prediction of demographic variables on the latent categories of college students’ lifestyle (*n* = 1340)

Model terms		Moderate Engagement		Negative Engagement	
		OR (95% CI)	*p*	OR (95% CI)	*p*
Sex					
Female		1.527 (1.174, 1.985)	0.002	1.163 (0.874, 1.547)	0.302
Family monthly income (RMB)					
< 2000		1.673 (0.935, 2.993)	0.083	2.937 (1.404, 6.144)	0.004
2000~5000		1.491 (0.821, 2.706)	0.189	1.920 (0.900, 4.094)	0.091
Smoking					
Yes		1.515 (0.557, 4.116)	0.416	1.971 (0.742, 5.234)	0.173
Alcohol use					
Yes		1.285 (0.488, 3.384)	0.612	2.024 (0.793, 5.169)	0.140

*CI* Confidence interval

### Associations between lifestyle behaviors and mental health

After adjusting for sex, age, family monthly income, smoking, and alcohol use, multiple linear regression analysis was conducted using lifestyle classification as the independent variable and the DASS-21 three dimensions as dependent variables. The classification results were set as dummy variables, with the “Active Engagement” group as the reference group, and the results are shown in Table 4. Compared with students in the “Active Engagement” group, those in the “Moderate Engagement” and “Negative Engagement” groups scored significantly higher on measures of depression, anxiety, and stress levels (all *p* < 0.001). Compared with students in the “Active Engagement” group, the mean scores for depression, anxiety, and stress in “Moderate Engagement” group were 1.274, 1.606, and 1.717 points higher, respectively. The increase was even more pronounced in the “Negative Engagement” group, with depression scores increasing by 10.253 points, anxiety scores by 9.912 points, and stress scores by 10.881 points. Moreover, the unhealthier the lifestyle, the greater the negative impact on mental health, as well as on the three dimensions of depression, anxiety, and stress. These results indicated that unhealthier lifestyle profiles were associated with progressively worse mental health outcomes across all three DASS-21 dimensions.

**Table 4:** Profiles of healthy lifestyle behaviour and risk of mental health (*n* = 1340)

Model terms		Model 1*		Model 2†	
		B (95% CI)	*p*	B (95% CI)	*p*
Depression					
Active Engagement		Ref.		Ref.	
Moderate Engagement		1.187 (0.666, 1.708)	< 0.001	1.274 (0.753, 1.795)	< 0.001
Negative Engagement		10.283 (9.711, 10.855)	< 0.001	10.253 (9.679, 10.827)	< 0.001
Anxiety					
Active Engagement		Ref.		Ref.	
Moderate Engagement		1.598 (1.082, 2.115)	< 0.001	1.606 (1.086, 2.127)	< 0.001
Negative Engagement		9.962 (9.395, 10.530)	< 0.001	9.912 (9.339, 10.485)	< 0.001
Stress					
Active Engagement		Ref.		Ref.	
Moderate Engagement		1.645 (1.096, 2.195)	< 0.001	1.717 (1.166, 2.268)	< 0.001
Negative Engagement		10.925 (10.321, 11.528)	< 0.001	10.881 (10.275, 11.488)	< 0.001

*95% CI* 95% confidence interval; * No adjustment; † Adjusted for sex, age, family monthly income, smoking and alcohol use

## Discussion

This study aimed to identify similar subgroups, or profiles, of individuals based on five lifestyle-related behaviors and to examine the association between these profiles and mental health in Chinese college students. Three distinct profiles were identified: “Active Engagement” (characterized by the highest PA, lowest sedentary behavior and screen time), “Moderate Engagement” (representing intermediate levels across behaviors), and “Negative Engagement” (characterized by the lowest PA, highest sedentary behavior and screen time). Monthly family income and sex were found to have a predictive effect on the classification of college students’ lifestyles. Significant differences in anxiety, depression and stress were observed among the three groups. The pronounced mental health risks associated with the “Negative Engagement” profile (characterized by insufficient PA and sedentary behavior) highlight the critical role of behavioral interventions in this population.

Multiple aspects of lifestyle can affect college students’ mental health [13, 22]. Crucially, our LPA approach allowed us to move beyond examining single behavior and instead focus on how clusters of co-occurring behaviors are associated with mental health outcomes (anxiety, depression, stress). LPA in this study identified three profiles, reflecting heterogeneity in students’ lifestyles. The combined proportion of “Active Engagement” and “Moderate Engagement” profiles was 74.5%, indicating that most students exhibit at least moderately healthy behaviors, consistent with prior research [23, 24]. Nevertheless, cultural and regional differences, such as the urban-rural gap and economic gradient, may result in different lifestyle patterns [25]. Therefore, multi-site research is needed to validate lifestyle patterns across different backgrounds.

The most striking finding were the significant differences in mental health observed across the three profiles. Individuals in the “Active Engagement” profile consistently exhibited the most favorable mental health status. Conversely, those in the “Negative Engagement” profile reported significantly higher levels of anxiety, depression, and stress, indicating substantially elevated mental health risks. The “Moderate Engagement” group’s mental health levels fell between the two aforementioned groups. These distinctions strongly suggest that high levels of PA, low sedentary time, and limited screen time – defining the “Active Engagement” profile – have a particularly beneficial impact on college students’ positive mental health. This finding aligns with growing evidence highlighting the unique mental health benefits of an active lifestyle [26, 27], specifically through the integration of physical activity while reducing prolonged sedentary behavior and excessive screen exposure, which positively affects college students’ mental health [28, 29]. These distinct profiles provide a practical framework for universities to implement targeted mental health promotion initiatives. Specifically, students identified with the “Negative Engagement” profile can be designed intensive, multi-behavioral interventions (e.g., combining activity incentives with screen time reduction strategies), while those in the “Moderate Engagement” group may benefit most from motivational support to transition towards the “Active Engagement” pattern. Furthermore, these profiles could be directly applied to digital screening tools or platforms to automatically categorize students based on behavioral data, facilitating timely and personalized feedback or resource allocation [30, 31].

It is worth noting that, compared to the significant differences in physical activity, sedentary behavior, and screen time, the differences in dietary behavior are not as pronounced. Although diet quality is undoubtedly important for mental health [32], our profile-based analysis suggests that, within the context of these identified lifestyle patterns, variations in diet may play a relatively less dominant role in differentiating mental health outcomes among these specific profiles. Although there are significant differences in mental health status between the “Active Engagement” and “Negative Engagement” profiles, the differences in diet are not as pronounced as those observed in movement-related behaviors. We speculate that for individuals with characteristics similar to those identified here, interventions primarily targeting increases in physical activity coupled with reductions in sedentary/screen time may yield more substantial and immediate mental health benefits than interventions focused solely on diet. However, diet is complex, and its effects may interact with other profile characteristics or change dynamically over the long term. Further research is needed to explore the combined effects of diet and other lifestyle factors on college students’ mental health.

There is a well-established association between socioeconomic status and mental well-being throughout the lifespan. In this study, participants with the lowest family income exhibited the highest risk of mental health disorders compared with those form higher income backgrounds, consistent with previous research findings [33]. Greater financial difficulties are associated with higher rates of depression, anxiety, and other mental health challenges [34], which may reflect ongoing concerns about educational costs, living expenses, and future financial obligations-factors that can have negative effects on mental and emotional well-being [35]. Although monthly household income was identified as a significant predictor, this may reflect limited statistical power to detect other influence factors or the presence of unexamined interactions, such as those involving sex or parental education. Future research should address these possibilities to clarify the complex interactions among socioeconomic factors in mental health profiles. Targeted interventions and further research are necessary to address these multifactorial challenges and enhance college student well-being.

Several limitations of this study should be acknowledged. First, participants were recruited from a single institution, which may constrain the generalizability of the findings. Future research should employ multi-center surveys across a broader range of universities and cultural backgrounds to improve external validity and verify profile transferability. Second, all data were self-reported and may not fully represent actual behaviors; for example, screen time may have been underestimated. Third, although major covariates were included, unmeasured confounders, such as genetic predisposition or peer influences may partly account for the observed associations. Fourth, although a minimum of three dietary recalls is required to accurately represent habitual diet, only one 24-hour dietary recall was used to calculate dietary diversity in our study, which may not fully reflect the habitual diet. Future studies will benefit from incorporating longitudinal dietary assessments (e.g., multi-day food diaries or repeated measures) to enhance ecological validity. Fifth, the absence of attention checks (e.g., instructional manipulation items) or perceived exertion scales limits our ability to detect unreliable responses in online self-reports. Future studies should implement such indicators to identify inattentive responding and enhance data validity. Finally, due to the cross-sectional design, causality cannot be established. Mental health may be either a consequence or a determinant of specific lifestyle behaviors. Further longitudinal and prospective studies are required to clarify these relationships.

## Conclusion

In summary, this study applied LPA to identify three profiles of lifestyle behavior among college students: “Active Engagement”, “Moderate Engagement”, and “Negative Engagement”. Students with “Active Engagement” profiles generally exhibited more favorable mental health status than those with “Negative Engagement”, as indicated by lower levels of anxiety, depression, and stress. Critically, our pattern-based approach revealed that the “Active Engagement” profile (characterized by the higher PA, low sedentary behavior and screen time) demonstrates unique importance for mental health - moving beyond simply promoting single behaviors. Given that college students experience a transitional period characterized by identity formation and increased autonomy, targeted strategies that promote healthier lifestyles may contribute to the maintenance of mental health in this population.

## Data Availability

No datasets were generated or analysed during the current study.

## Abbreviations

DQQ: Diet Quality Questionnaire
ISI: Insomnia Severity Index
PA: Physical activity
DASS-21: Depression, Anxiety and Stress Scale-21
LPA: Latent Profile Analysis
IPAQ-SF: International Physical Activity Questionnaire Short Form
AIC: Akaike Information Criterion
BIC: Bayesian Information Criterion
a-BIC: Adjusted BIC
LMR-LRT: Lo-Mendell-Rubin-likelihood ratio test
BLRT: Bootstrapped-Likelihood Ratio Test
